# Green synthesis, characterization, and antibacterial activity of *Citrus lanatus* based silver nanoparticles

**DOI:** 10.6026/97320630019403

**Published:** 2023-04-30

**Authors:** Aravinthrajkumar Govindaraj, Mahesh Ramakrishnan, Rajeshkumar Shanmugam, Saravana Dinesh SP

**Affiliations:** 1 Department of Orthodontics, Saveetha Dental College and Hospitals, Chennai-600077

**Keywords:** Green synthesis, antibacterial, *Citrus lanatus*, silver nanoparticles

## Abstract

Due to their enhanced and unique physicochemical characteristics, such as their minimal dimensions, large surface area compared to their mass and increased reactivity, nanomaterials hold promise in the field of antibacterial therapy. The aim of this study is to evaluate the method for Green Synthesis, Characterization of *Citrus lanatus* pulp, rind, and seed-based Silver Nanoparticles, and study its antibacterial activity against common oral microorganisms. Green synthesis of nanoparticles was formulated using the extract from the rind, seed, and pulp of *Citrullus lanatus* and silver nitrate extract. The extract was stirred in a magnetic stirrer for 24 hours, and centrifugation was done at 8000 rpm for 10 minutes. The synthesized nanoparticles were characterized and utilized for the antibacterial study. The characterization was done using UV-visible light spectroscopy, and antibacterial activity was assessed using the well diffusion method and measuring the zone of inhibition. The nanoparticles synthesized were characterized using UV-visible spectroscopy, and the spectroscopic analysis confirmed the formation of silver particles in this study. At 450 nm, a sharp peak was observed, which correlated to the SPR band of the particles. The results showed that the zone of inhibition assessed was comparable to the standard. From this study it can be concluded that silver nanoparticles (AgNPs) produced by green synthesis from *Citrullus lanatus* showed significant antibacterial potential against common oral microflora.

## Background:

Nanotechnology is a branch of science that deals with nanoparticles, or things that are smaller than a nanometer (NPs). Nanomaterials are tiny solid particles with a size between one and one hundred nanometers. Due to their enhanced and unique physicochemical characteristics, such as their minimal dimensions, large surface area compared to their mass and increased reactivity, nanomaterials hold promise in the field of antibacterial therapy. [1] The availability of a wide variety of metabolites with strong reduction potentials, global distribution, safe handling, minimal waste and energy costs, large and accessible reserves, and widespread use of plant extracts are only a few of the reasons why they are so popular. [2] Due to several features, including a variable surface area-to-volume ratio that is useful in a variety of biological and technological applications, silver nanoparticles (AgNPs) have received commercial attention. Their biological activity in relation to antibacterial, anticancer, and antioxidant effects in medical settings have been assessed in various studies.[3, 5] In comparison to numerous antibacterial compounds, Silver(Ag) NPs have demonstrated a very high antimicrobial action with good biocompatibility. NPs have also been employed as the primary component of antibacterial nan ocomposites based on inorganic and polymeric materials.[6] There are physical, chemical, and biological ways to make silver nanoparticles (NPs). In the top-down approach, bulk metals are mechanically ground, and the resulting nanosized metal particles are then stabilized by the addition of colloidal stabilizers. Contrarily, the bottom-up approaches use sono decomposition, electrochemical techniques, and metal reduction. The simplest approach involves reducing the metal salts AgBF4 chemically with NaBH4 in water. The NPs that are produced in this manner range in size from 3 to 40 nm.[7, 8, 9] Utilizing microorganisms like fungi, yeasts (eukaryotes), bacteria, and actinomycetes (prokaryotes), using plants and plant extracts, or using templates like membranes, viruses' DNA, and diatoms are all examples of green synthesis. [10] In this original article, we are going to see the method for green synthesis, the characterization of Citrus lanatus pulp, rind, and seed-based silver nanoparticles, and study their antibacterial activity against common oral microorganisms.

## Materials and Methodology:

The study was conducted in the department of pharmacology and nanotechnology at the university after getting approval from the institutional scientific committee and the scientific review board. This is an in vitro study that was conducted for the green synthesis of silver nanoparticles from the rind, pulp, and seed extract of *Citrullus lanatus*

## Preparation of *Citrullus lanatus* extracts:

A fresh-cut pulp of *Citrullus lanatus* was cut using a sterile knife. The seeds were removed from the cut pieces, and the pulp was crushed using a mortar and pestle to obtain 100 ml of pure extract. The obtained watermelon juice was filtered using sieves, and secondary filtration was done using Whatman grade 1 filter paper. To obtain the extract of the seeds and rind of *Citrullus lanatus*, the seed and rind were cut and dried in a hot air oven. The dried seed and rind of *Citrullus lanatus* were powdered, and the extract was prepared using distilled water. The amount of material used in the extract's synthesis can be changed depending on the amount of extracted particles required.

## Synthesis of Nanoparticles:

**Figure 1:**
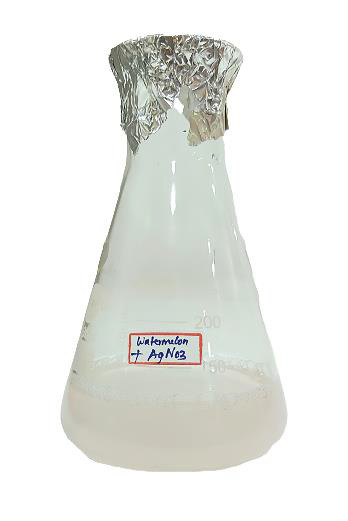
Silver nitrate (AgNO3) mixed with *Citrullus lanatus* extract.

Green synthesis of silver nitrate nanoparticles weighing 0.0169 g dissolved in 90 mL distilled water to produce a pure extract of silver nitrate. Fresh *Citrullus lanatus* extract was added to that 10 mL and stirred in a magnetic stirrer for 24 hours (Figure 1). After which, centrifugation was done at 8000 rpm for 10 minutes. The supernatant was discarded, and the pellet was stored in an airtight Eppendorf tube at 4 degrees Celsius. The synthesized nanoparticles were characterized and utilized for the antibacterial study. The quantity of the material used in the synthesis of nanoparticles and the duration taken to prepare them can be altered and will change, respectively, depending on the quantity of the nanoparticle required.

## Characterization of nanoparticles:

UV-vis spectroscopy was used to assess the produced nanoparticles' UV-vis absorption peak. The chosen scanning range is 330-660 nm. The transformation of *Citrullus lanatus* with silver nitrate solution into AgNPs is necessary for UV-vis spectroscopic investigation. The appearance of a shift in hue will signal the creation of NPs.

## Antibacterial activity:

The antibacterial activity of the extracted *Citrullus lanatus* based silver nanoparticles was assessed against common oral bacteria such as *S. aureus*, *S. mutans*, *C. albicans*, *E. faecalis*, and *Lactobacillus sp*. For this experiment, MHA agar was used to identify the zone of inhibition. Prepared Muller-Hinton agar was sterilized for 45 minutes at 120 lbs. Sterilized plates were filled with the medium, which was then left to solidify. The test organisms were swabbed after the wells were cut with the well cutter. Three different sources of nanoparticles were added, keeping chlorhexidine as the standard, and the plates were incubated for 24 hours at 37°C. The zone of inhibition was assessed following the incubation period.

## Results:

## Ultraviolet–visible spectroscopy:

The surface plasmon resonance (SPR) shows an unusual occurrence of noble metal nanoparticles that generate intense electromagnetic fields on the surface of the particles, and thus the radioactive properties such as absorption and scattering are increased. Hence, UV spectroscopic analysis confirmed the formation of silver particles in this study. At 450 nm, a sharp peak was observed, which correlated to the SPR band of the particles.

## Antibacterial activity:

**Figure 2:**
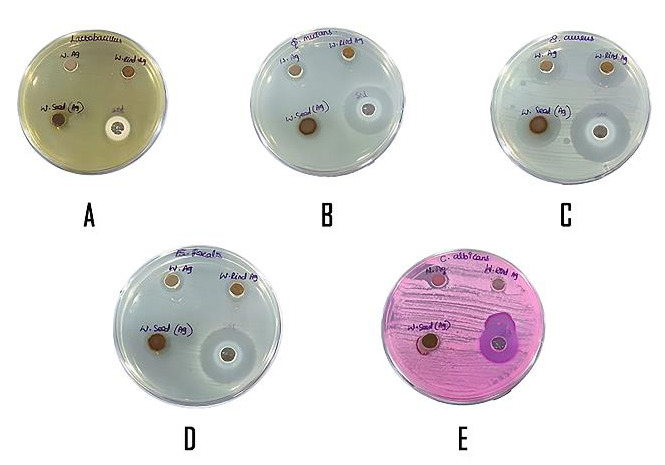
Zone of Inhibition; A - Antimicrobial effect of *Citrullus lanatus* based silver nanoparticles on *Lactobacillus sp*.; B - Antimicrobial effect of *Citrullus lanatus* based silver nanoparticles on *S. mutans*.; C - Antimicrobial effect of *Citrullus lanatus* based silver nanoparticles on *S. aureus*.; D - Antimicrobial effect of *Citrullus lanatus* based silver nanoparticles on *E. faecalis*.; E - Antimicrobial effect of *Citrullus lanatus* based silver nanoparticles on *C. albicans*.

The antibacterial activity of the synthesized nanoparticles was studied by assessing the zone of inhibition (Figure 2). The zone of inhibition measured using the vernier caliper is given in the table below (Table 1). The zone of inhibition showed that there was significant antibacterial activity against all the bacteria. The antibacterial activity was increased in seed NPs when compared to that of pulp and rind NPs, and the antibacterial activity was significantly higher against Staphylococcus aureus than the other bacteria. The antibacterial activity of all the nanoparticles was comparable to that of the standard, chlorhexidine.

## Discussion:

The findings of this study demonstrated that Ag nanoparticles, green synthesized from *Citrullus lanatus*, have a significant antibacterial effect against common oral bacteria like *S. mutans*, *S. aureus*, *C. albicans*, *Lactobacillus sp*., and *E. fecalis*. AgNPs are a promising system with key characteristics including antibacterial, anti-inflammatory, and anticancer action, according to research. They may also be used as carriers in sustained drug delivery. They said that more research was needed to fully understand some elements of the processes by which AgNPs work, as well as some significant toxicological issues brought on by the usage of this technology. [11, 12] Researchers looked at the antibacterial effects of AgNPs added to dental materials such as composite resin, endodontic materials, acrylic resin, and implants. Additionally, numerous studies have demonstrated that silver, in its nanoparticulated form, has an inhibitory effect against a variety of bacteria and fungi, including *S. mutans*, *C. albicans*, *P. aeruginosa*, *E. faecalis*, and *S. aureus*, among others. This could reduce the risk of secondary caries, fungal infections, failed endodontic treatments, and dental implants.[13, 14] The antibacterial activity of *Citrullus lanatus* has been studied earlier in various studies earlier. The study on the antibacterial activity of watermelon extract against oral microflora showed that there was a significant effect against *Lactobacillus*[15]. The activity of various components of *Citrullus lanatus* was studied in various articles and showed that all the components of it, including the rind, pulp, and seed, showed significant antibacterial activity against oral microflora like *Streptococcus mutans*, *Staphylococcus aureus*, *Lactobacillus sp*., *Candida albicans*, and *E. fecalis*.[16, 17, 18] The UV spectroscopy method was used to characterize the nanoparticles synthesized. Numerous studies have shown the use of a UV-Vis spectrophotometer to track the bio-reduction of silver ions in aqueous solutions. In these investigations, pure Ag+ ion reduction was often observed 3-5 hours after a tiny aliquot of the sample was diluted in distilled water. [19, 22] Another study on the green synthesis of silver Ag nanoparticles came to the conclusion that these particles might be used as antibacterial agents. The characterization research demonstrated that the particles created in nano dimensions would be just as useful in therapeutic formulations as antibiotics and other medications. [23, 24, 25]

## Conclusion:

Thus, silver nanoparticles (AgNPs) produced by green synthesis from *Citrullus lanatus* rind, fruit, and seeds, showed significant antibacterial potential against common oral microflora. The antibacterial activity assessed by using the zone of inhibition was increased with seed extract AgNPs when compared with fruit and rind extract silver nanoparticles.

## Clinical Significance:

By lowering the quantity of common oral bacteria that cause carious lesions, these newly created silver nanoparticles based on *Citrullus lanatus* can be employed in the indirect prevention and creation of new dental caries.

## Limitations:

This in-vitro study compares the antibacterial effectiveness of silver nanoparticles derived from *Citrullus lanatus* to the industry standard of chlorhexidine. To confirm the study's findings, more research utilizing human clinical trials is essential.

## Funding:

There is no source of funding.

## Figures and Tables

**Table 1:** Zone of Inhibition

**Bacteria sp.**	**Pulp NPs**	**Rind NPs**	**Seed NPs**	**Standard**
*Lactobacillus sp.*	9	10	14	18
*S.mutans*	14	16	18	22
*S.aureus*	22	26	29	32
*E.fecalis*	14	16	18	22
*C.albicans*	12	10	11	16
